# Editorial: MRI-Based Methods for the Identification of Cerebellar Ataxia Types

**DOI:** 10.3389/fnins.2022.847726

**Published:** 2022-02-16

**Authors:** Salem Hannoun, Roula Hourani

**Affiliations:** ^1^Medical Imaging Sciences Program, Division of Health Professions, Faculty of Health Sciences, American University of Beirut, Beirut, Lebanon; ^2^Department of Diagnostic Radiology, American University of Beirut Medical Center, Beirut, Lebanon

**Keywords:** ataxia, markers, neurodegeneration, MRI, detection

Failure to coordinate movements in ataxia patients results in gait-limb ataxia, frequent falls, dysarthria, and oculomotor abnormalities such as nystagmus or saccadic dysmetria (Palau and Espinós, 2006). Lesions' location in either of the cerebellum's parts might cause a distinct sort of ataxia. For instance, gait and truncal ataxia are caused by damage to the midline cerebellar regions, whereas ipsilateral limb cerebellar ataxia is caused by damage to the unilateral cerebellar hemisphere (Ashizawa and Xia, 2016).

The strength of neuroimaging in diagnostic investigations is currently based in part on pattern-recognition techniques comparable to those employed in brain tumors. Two separate trends of cerebellar ataxia might be found based on neuroimaging data: degenerative or malformative. Cerebellar atrophy characterizes the degenerative pattern, which is frequently coupled with white matter (WM) or gray matter (GM) T2/FLAIR signal alterations, whereas aberrant morphology of the brain stem and/or cerebellum characterizes the malformative pattern (Vedolin et al., 2013). For example, in Friedrich's ataxia (FRDA), the cerebellum volume is retained despite modest upper vermis atrophy, reduced dentate nuclei, and higher iron accumulation (Barbeau, 1978; Schipper, 2012; Stefanescu et al., 2015). The vermis is generally atrophied and typically isolated in young children with ataxia-telangiectasia (AT) (Perucca et al., 2016). The iron-induced signal ordinarily seen in dentate nuclei vanishes in ataxia with oculomotor apraxia types 1 and 2, and widespread cerebellar atrophy predominates in the anterior vermis (Frismand et al., 2013). Mild vermis and cerebellum atrophy, as well as volume reduction of the dentate nuclei and atrophy of the middle cerebellar peduncle, have been documented in spinocerebellar ataxia type 3 (SCA3) (Eichler et al., 2011; Jacobi et al., 2012). The vermis and cerebellar hemispheres showed significant atrophy in SCA6, whereas the middle cerebellar peduncle showed no signs of atrophy (Eichler et al., 2011; Jacobi et al., 2012).

This Research Topic includes four original research articles, one brief research report, and one systematic review that examine the radiological features of distinct forms of cerebellar ataxias depending on their categorization and etiologies. Wang et al. used the 3D fractal dimension approach to measure morphological alterations in supratentorial areas and estimate atrophy in relatively focal regions of 48 SCA3 patients and 50 healthy controls (HC). Their results showed that SCA3 atrophy is not only limited to infratentorial regions. In fact, both cerebellum and basal ganglia related cortex were impacted. These findings might be linked to common SCA3 symptoms and suggest that SCA3 should no longer be thought of as a disease affecting only the cerebellum and its connections, but rather as an illness impacting the whole brain.

Vavla et al. longitudinally evaluated advanced MRI and retinal imaging techniques in 11 FRDA patients in a study testing the safety and efficacy of 6-month treatment with interferon gamma. While the diffusion tensor imaging indices showed a gradual reduction of fractional anisotropy, functional MRI (fMRI) and resting-state fMRI (rs-fMRI) showed substantial changes during and after therapy. The changes in fMRI were shown to have a strong relationship with clinical response. The retinal nerve fiber layer thickness was known to be thinner on optical coherence tomography, but there was no change over time. This pilot study suggests that fMRI and rs-fMRI might be useful as auxiliary measures in clinical trials for FRDA.

In their systematic review, Vavla et al. also gave a critical assessment of the findings and techniques of fMRI investigations undertaken in genetically proven FRDA. A total of 198 FRDA children and young adults, and 205 HC were enrolled, in 12 cross-sectional and longitudinal fMRI studies. Motor and cognitive task paradigms, as well as resting-state investigations, were among the reported fMRI designs, with broad alterations in functionally engaged regions and a wide range of study approaches. These studies provided a mixed picture of hypo- and hyperactivations in distinct cerebral and cerebellar areas. Clinical factors and functional changes were also frequently linked. Overall, the data support the idea of cerebro-cerebellar loop injury and compensatory mechanisms.

The goal of Nigri et al. was to determine when early clinical and neurodegenerative MRI alterations may be detected, and assess the rate of disease progression in both preclinical and early disease stages. In their one-year longitudinal study, 14 SCA2 patients, 13 presymptomatic SCA2 participants (preSCA2), and 15 HC were recruited. SCA2 patients had significant atrophy in the cerebellum, brainstem, basal ganglia, and cortex, whereas preSCA2 subjects had isolated volume loss in the pons, as well as cortical thinning in specific frontal and parietal areas, such as the rostral-middle-frontal and precuneus. The one-year follow-up showed volume loss in the cerebellum, pons, superior cerebellar peduncles, and midbrain in SCA2 patients, but primarily in the cerebellum in preSCA2 participants. This pilot study showed that MRI measures are very sensitive in detecting longitudinal structural changes in SCA2 patients, as well as in preSCA2 individuals up to a decade before projected illness onset.

Alata et al. presented a longitudinal investigation of alterations in the cerebellum, corpus callosum (CC), ventricular system, and striatum of an H-ABC (Hypomyelination with atrophy of the basal ganglia and cerebellum) patient and *taiep* rat. They compared the patient's MRI findings to the results of immunofluorescence, gait analysis, cerebellum, CC, and ventricular system segmentation in the *taiep* rat. They discovered that cerebellar and callosal alterations, which might indicate hypomyelination, deteriorated with age and coincided with the onset of ataxic gait. They also reported an increased lateral ventriculomegaly in both the patient and *taiep*, perhaps due to WM degeneration. These WM alterations progress over time and may contribute to clinical deterioration.

Lupo et al. assessed distinct MRI patterns that might be associated with Spastic Paraplegia 7 (SPG7) mutations and may be linked to patients' cognitive profiles in six SPG7 individuals and 30 HC. MRI voxel-based morphometry and functional connectivity methods were used to assess the cerebello-cortical network. In parallel, the cognitive and social functioning of SPG7 patients was examined. Their findings revealed particular changes in language, verbal memory, and executive function, as well as impairments in social and emotional processes. The presence of cerebello-cortical dysregulation in different networks involved in cognition and social functioning in SPG7 patients is confirmed by evidence of an over-connectivity pattern between both the right and left cerebellar dentate nuclei and specific cerebral regions.

## Author Contributions

SH wrote the first draft. RH provided critical comments and editorial suggestions for revisions. Both authors contributed to the article and approved the submitted version.

## Conflict of Interest

The authors declare that the research was conducted in the absence of any commercial or financial relationships that could be construed as a potential conflict of interest.

## Publisher's Note

All claims expressed in this article are solely those of the authors and do not necessarily represent those of their affiliated organizations, or those of the publisher, the editors and the reviewers. Any product that may be evaluated in this article, or claim that may be made by its manufacturer, is not guaranteed or endorsed by the publisher.
